# Cell adhesion and mechanical stimulation in the regulation of mesenchymal stem cell differentiation

**DOI:** 10.1111/jcmm.12061

**Published:** 2013-05-15

**Authors:** Yang-Kao Wang, Christopher S Chen

**Affiliations:** a Graduate Institute of Biomedical Materials and Tissue Engineering, Taipei Medical UniversityTaipei, Taiwan; b Center for Neurotrauma and Neuroregeneration, Taipei Medical UniversityTaipei, Taiwan; c Department of Bioengineering, University of PennsylvaniaPhiladelphia, PA, USA

**Keywords:** microenvironment, cell adhesion, mechanical force, mesenchymal stem cell, differentiation

## Abstract

Stem cells have been shown to have the potential to provide a source of cells for applications to tissue engineering and organ repair. The mechanisms that regulate stem cell fate, however, mostly remain unclear. Mesenchymal stem cells (MSCs) are multipotent progenitor cells that are isolated from bone marrow and other adult tissues, and can be differentiated into multiple cell lineages, such as bone, cartilage, fat, muscles and neurons. Although previous studies have focused intensively on the effects of chemical signals that regulate MSC commitment, the effects of physical/mechanical cues of the microenvironment on MSC fate determination have long been neglected. However, several studies provided evidence that mechanical signals, both direct and indirect, played important roles in regulating a stem cell fate. In this review, we summarize a number of recent studies on how cell adhesion and mechanical cues influence the differentiation of MSCs into specific lineages. Understanding how chemical and mechanical cues in the microenvironment orchestrate stem cell differentiation may provide new insights into ways to improve our techniques in cell therapy and organ repair.

- Introduction
- General introduction of stem cell biology
- Cell adhesion and the generation of adhesion forces
- Cell adhesion regulates MSC differentiation
- Substratum stiffness and MSC fate decisions
- Molecular mechanisms relaying substratum stiffness-regulated cellular responses
- Mechanical forces control MSC differentiation
- Summary
- Conclusions and future prospects

## Introduction

Many patients suffer from chronic organ failure, brought about by myocardial infraction, chronic renal failure, diabetes, neural degenerative diseases, etc. The major problem in these chronic diseases is the progressive damage to tissues combined with the absence of adequate endogenous repair systems. Tissue function is gradually lost, and the patient's condition becomes critical. Currently, allogenic organ transplantation is the preferred approach for restoration of organ function. Despite attempts to encourage organ donation, however, there is a shortage of transplantable human tissues and organs worldwide. Another disadvantage of organ transplantation is the serious outcome of organ rejection by recipients. For these reasons, researchers are investigating new approaches for treating organ failure. An alternative to organ or tissue transplantation is the use of cell-based therapies and tissue engineering to circumvent the shortage of organ donors. Over the past few years, several studies reported results that show great potential for cell-based therapy in regenerative medicine 1–3. One of the major limitations of cell-based therapy is the limited number of available human cells. Another problem is when isolated and expanded *in vitro*, several cell types dedifferentiate and lose their specific differentiation characters that are necessary for their function. Recently, stem cells isolated from either developing embryos or adult human tissues or genetically reprogrammed from adult cells have been shown to be a superior source of undifferentiated progenitor cells. With the importance of stem cells in tissue engineering becoming well recognized, researchers are currently focusing upon regulating stem cell commitment and applying stem cells to tissue engineering.

## General introduction of stem cell biology

Stem cells are defined as unspecialized precursor cells capable of self-renewal and differentiation into diverse specialized cell lineages under appropriate stimuli. Stem cells can be categorized into three broad types based on their ability to differentiate. Totipotent cells are derived from early embryos, in which each cell can form a new individual. Pluripotent stem cells [*e.g*. embryonic (E)SCs] are derived from the undifferentiated inner cell mass of the blastocyst in early embryos and can form most of the cell types in the body except the placenta. Although ESCs are an excellent source for generating different cell types, the experimental design of applying ESCs to tissue replacement is poorly understood. ESCs have been used in several degenerative disease models and may provide better treatment outcome for those diseases 4–6. There are still several obstacles to be overcome, for example, after ESCs are transplanted into tissues, they give rise to teratomas, which can eventually kill the host. Thus, preventing ESCs from outgrowth and controlling the ESC fate to obtain specific types of tissues are some of the major challenges in the stem cell biology. Developing treatments for these diseases will require better knowledge of the pathways, which are necessary for uncommitted cells to become differentiated and restore the function of damaged tissues. Mesenchymal stem cells (*e.g*. MSCs) are derived from foetal tissues, cord blood, placenta and other adult tissues. Despite the ability of these cells to differentiate into other lineages being more limited than pluripotent stem cells, these multipotent cells already have a track record of success in cell-based therapies 7–9. Human (h)MSCs isolated from bone marrow aspirates have been shown to differentiate into fabricated bone, cartilage, fat, muscles, tendons/ligaments and other connective tissues both *in vivo* and *in vitro* 10, 11. Transplantation of bone marrow–derived stem cells into adult mice can give rise to brain astrocytes 12 and neurons 13. It was also been reported that MSCs can undergo transdifferentiation, in which mesenchymal cells are induced to become parenchymal cell types, such as hepatocytes 14, 15, endothelial cells 16 or cardiomyocytes 17. In addition to their differentiation ability, isolated MSCs are able to grow in the presence of foetal bovine serum and maintain the potential to differentiate into different lineages. However, the two important features of self-renewal and multipotentiality of stem cells become limited when these cells are introduced into *in vitro* culture, and MSCs progressively senescence 18, 19. In particular, long-term culturing on rigid substrata inevitably leads to decreased growth rates and eventual senescence, with concomitant decreases in the differentiation propensity and telomere length 20, 21. In addition, adult stem cells exhibit significant donor-to-donor variability in proliferation rates and differentiation potential 18, 22, 23. These phenomena are critical because therapeutic tissue engineering requires large and reliable production of donor-specific cells. It is important to be able to induce MSC proliferation without losing the differentiation potential both *in vivo* and *in vitro*.

Various growth factors and cytokines have been applied to study the control of stem cell proliferation. Treatment of hMSCs with fibroblast growth factor (FGF)-2 resulted in a faster proliferation rate, and the doubling time was always shorter than the untreated controls 24. Incubation of MSCs with bone morphogenetic protein (BMP)-2 significantly increased proliferation as seen by bromo-deoxyuridine (BrdU) incorporation, cell cycle progression and the expression of proliferating cell nuclear antigen (PCNA) 25. Cell proliferation was further enhanced by the combined treatment with BMP-2 and FGF-2, possibly because of synergistic effects resulting from signal crosstalk between these two different stimuli 26. Both BMP-2 and FGF-2 are also involved in inducing osteogenesis of MSCs 26. Other than these soluble factors, recent studies also demonstrated that several extracellular matrix (ECM) proteins are able to preserve the proliferation and differentiation potential of MSCs. The ECM made of bone marrow cells facilitates expansion of MSCs 27, 28. This ECM is most likely composed of basement membrane proteins, including collagen types I, III and V, syndecan-1, perlecan, fibronectin, laminin, biglycan and decorin 27. In addition, treatment with hyaluronan alone can also preserve the proliferation and differentiation potential of MSCs 29. Those studies suggested that appropriate control of the stem cell growth and its stemness by these environmental cues may provide insights into how to maintain and expand stem cell for *in vitro* culture systems.

## Cell adhesion and the generation of adhesion forces

Cells adhere to the ECM through specific classes of transmembrane receptor integrins. Binding of integrins to the ECM causes their clustering in cell membranes 30, which in turns leads to the recruitment of focal adhesion proteins that participate in intracellular signalling pathways or that mechanically connect integrins to the cytoskeleton 30, 31. The assembly and disassembly of focal adhesions are very highly regulated and play critical roles in cell spread and migration 32–36. Focal adhesions evolve from small, dot-like structures located at the periphery of a spreading cell or the leading edge of a migrating cell, termed as focal complexes. These structures are nascent and can mature into focal adhesions 37. Apparently, because of the differentiation, localization, and size of focal complexes and focal adhesions, the actin cytoskeleton associated with them differently. The tensile force generated by actin filaments attached to focal complexes may also differ in magnitude from that of actin filaments attached to mature focal adhesions. Several studies have revealed that during the maturation of focal complexes to focal adhesions, both small guanine triphosphatase (GTPase) Rho and myosin light-chain kinase have been shown to regulate contractile forces of the actin cytoskeleton and formation of focal adhesions 38, 39. A decrease in myosin II–driven contractility has been shown to diminish the size of focal adhesions 40, and blocking contractility leads to complete dissolution of focal adhesions 32, 41. These studies suggest that the mechanisms of assembly and disassembly of focal adhesions are regulated by biochemical signals, and also by forces generated by actino-myosin contractions.

**Fig. 1:**
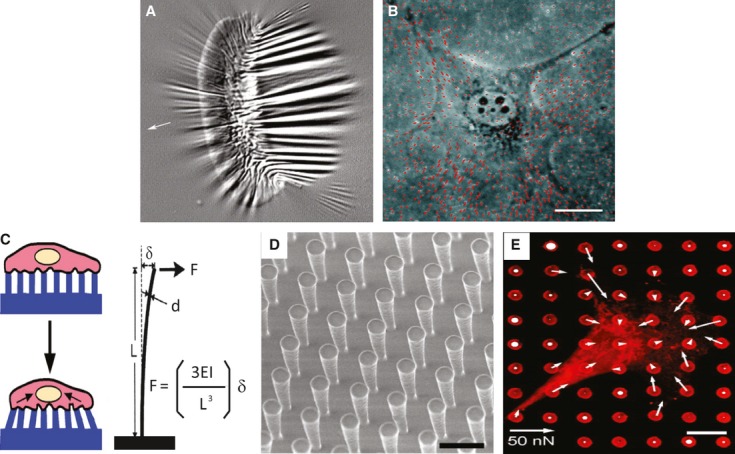
Tools for measuring cellular forces. (**A**) Fish keratinocytes cultured on a flexible silicon substrate and wrinkling of the film because of the generation of traction in cells. (**B**) 3T3 cells cultured on polyacrylamide (PA) gel embedded with fluorescent microbeads. Both A and B are reproduced with permission from Ref. 44. (**C**) Schematic illustration of cells lying on posts and deformation of the posts by exertion of traction force on the posts. (**D**) A uniform vertical microfabricated elastomeric array of posts. (**E**) Quantification of the subcellular distribution of traction forces. The length of the arrow indicates the magnitude of the calculated force. C, D and E were reproduced with the permission from Ref. 48.

Despite intensive efforts to understand how the cytoskeleton responds to chemical stimuli, the mechanisms by which forces are generated across cell surfaces and transduced into a cytoskeletal response are still poorly understood. Measuring the force that is generated at a focal adhesion is not a simple task. Spatial and temporal variations in force generated at focal adhesions from site to site make it challenging to precisely measure. Previous studies have successfully demonstrated measurement of forces in focal adhesions of cells cultured on flexible substrata, such as silicone membranes (Fig. 1A) 42. Deformation of a flexible substratum by cell-generated forces can be visualized by microscopy, and subsequently, lateral deformation of the substratum can be used to calculate local forces. However, silicon film does not behave like an ideal spring, and the complexity of the preparation procedures renders it difficult to use. An alternative flexible substratum for force measurements is polyacrylamide (PA) gel. PA gel has several advantages of easy preparation and superior mechanical properties. The flexibility of acrylamide gels can be easily controlled by simply adjusting the ratio of acrylamide to bis-acrylamide 43, and the three-dimensional (3D) porous structure mimics physiological conditions. Using displacements of embedded fluorescent beads, deformations of PA gels can be used to calculate the contractility (Fig. 1B) 43, 44. Through this approach, a linear relationship was found between the forces exerted at adhesion and the size of focal adhesions. Although these approaches provide strong correlations between the mechanical force and cell behaviour, these methods can neither provide causal relationships between forces and cellular behaviours nor offer appropriate detection of forces in all indicated intracellular regions. Recently, soft-lithography technology, derived from the semiconductor industry, has been used to control cell–ECM and cell–cell adhesions 45–47. A device, composed of microneedle arrays (posts) fabricated in a polydimethylsiloxane (PDMS) elastomer using a photolithographic method, was used to measure forces generated by spreading cells (Fig. 1C–E) 48. With application of microcontact printing, contractile forces of cells attached to different-sized areas can easily be quantified and compared. This device provides a better way to study both spatial and temporal changes in contractile forces generated by cells in response to environmental changes.

## Cell adhesion regulates MSC differentiation

It is now well accepted that MSC differentiation and phenotypic expression can be influenced by cues from the surrounding environment, both soluble (*e.g*. cytokines and growth factors) and insoluble (*e.g*. ECM density and stiffness) factors. However, the process of MSC differentiation is not fully understood. It has been well established that soluble growth factors (*i.e*. cytokines) regulate MSC commitments into different lineages 49–52. Although much effort has intensively been focused on soluble factors in MSC differentiation, little is known about the importance of cell adhesion in regulating MSC differentiation. In adipogenic differentiation, for example, changes in cell morphology, a decrease in assembly of cytoskeletal proteins and an increase in the activities of numerous lipogenic enzymes were found to be correlated with adipogenesis 53. In the context of adipogenesis, expressions of ECM receptor integrins are differentially regulated during adipogenesis. The level of α5 integrin gradually diminishes during the induction of adipogenesis, whereas that of α6 increases. Overexpression of α5 integrin results in enhanced proliferation and attenuated adipogenic differentiation, whereas overexpression of α6 integrin does not affect adipogenesis 54. Given that adipogenesis is a multistep process, one of the onsets of adipogenesis is ECM remodelling, as characterized by the conversion of the fibronectin-rich matrix into laminin-rich ECM 55–57. This result is consistent with data showing that fibronectin increases cell spreading and pre-adipocyte proliferation, while inhibiting adipogenic differentiation 53. Furthermore, cell adhesion and spread reflect cytoskeletal tension. In this context, changes in a cell's shape and cytoskeletal tension have been reported to be crucial in determining MSC lineage commitment into adipogenesis or osteogenesis 58. Spreading facilitates osteogenesis, whereas unspreading facilitates adipogenesis. Inhibition of cell spreading and cytoskeletal tension can also attenuate BMP-induced osteogenic differentiation in hMSCs 59. These results suggest that adhesion of cells to the ECM induces assembly of the actin cytoskeleton, which, through increasing spreading and cytoskeleton-driven tension, prevents adipogenic differentiation, but facilitates osteogenesis.

In addition to adipogenesis, a previous study has shown that blocking ECM–ligand interactions by applying the functional-perturbing anti-α5β1 integrin reduced both bone-like nodule formation and expressions of osteogenic genes. This result suggests that the α5β1 integrin mediates the binding of osteoblasts to fibronectin and is required for osteogenic differentiation 60 and suggests a pivotal role of fibronectin in osteoblast differentiation. Another study has demonstrated that even though hMSCs can adhere to various ECM-coated substrates (collagen I, fibronectin, vitronectin and collagen IV), the greatest osteogenic differentiation occurs among cells plated on vitronectin and collagen I 61. The type I collagen or α2β1 integrin recognition sequence Gly-Phe-Hyp-Gly-Glu-Arg (GFOGER) from the alpha [I] chain of type I collagen promotes activation of focal adhesion kinase (FAK), alkaline phosphatase and expression of osteogenic genes in murine pre-osteoblast-like cells 62. Blocking the interaction of type I collagen with its receptor α2β1 integrin by Asp-Gly-Glu-Ala (DGEA), an amino acid domain of type I collagen that interacts with the α2β1 integrin receptor on cell membranes, shows that the expression of the osteogenic phenotype of bone marrow–derived stromal cells is suppressed 62, 63. On the other hand, when hMSCs are plated on laminin-coated dish, the proliferation of hMSCs is more rapid than control cells, but the differentiation potential is still maintained. However, laminin suppresses chondrogenic differentiation of hMSCs, but has no effect on osteogenesis, suggesting that laminin may contribute to osteogenic differentiation by promoting proliferation and suppressing chondrogenic differentiation 64.

Other than differentiating into known mesenchymal tissues, investigators have also been working on inducing MSCs into neuronal lineages. Under certain conditions, MSCs can be induced to exhibit neuronal morphology and express protein markers that are typical of neurons 65, 66. It has also been shown that MSCs exhibit high proliferation on ECM-coated substrata that also support neuronal differentiation 67. These results indicate that multiple ECM proteins may provide a suitable environment for MSC attachment to the underlying substratum. The adhesion signal from each type of ECM protein is transmitted through a specific integrin to regulate differentiation. Although the aforementioned studies provided great insights into how adhesion regulates intracellular forces, whether or not mechanical stress mediates adhesion-induced MSC differentiation is still poorly understood.

## Substratum stiffness and MSC fate decisions

**Fig. 2:**
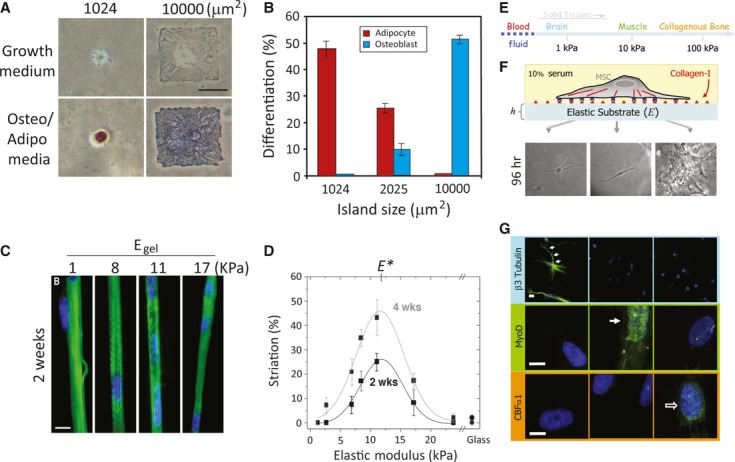
Mechanical stimulus–induced differentiation. (**A**) Cell shape drives mesenchymal stem cell (MSC) lineage commitment. Human (h)MSCs became bone only on large micropatterned islands, whereas adipogenesis occurred on small islands. (**B**) Quantitative results of MSC commitment on different-sized islands. Both A and B were reproduced from Ref. 58. (**C**) Myocytes cultured on collagen-coated polyacrylamide (PA) gels with various stiffness levels. Striated myotubes formed only on gels of intermediate stiffness. (**D**) Quantification results of optimal myotube formation on gels with different stiffness levels. Both C and D were reproduced from Ref. 80. (**E**) The elastic modulus of solid tissues. (**F**) The stiffness of the PA gel system can be modulated by changing the amount of the crosslinker. Cell adhesion to the gel can be controlled by covalent attachment of extracellular matrix (ECM) proteins (in this case, type 1 collagen). Human mesenchymal stem cells (hMSCs) seeded onto PA gels with different stiffness levels showed different morphologies. Cells were unspread with a branched morphology on soft substrate (0.1–1 kPa), had a bipolar morphology on intermediate stiffness (8–17 kPa) and had a polygonal morphology on stiff substrate (25–40 kPa) 96 hrs after seeding. (**G**) hMSCs differentiated into a neuronal lineage on soft substrate (0.1–1 kPa; as indicated by staining of βIII tubulin staining in cell branches); myogenic on intermediate stiffness (8–17 kPa; as indicated by MyoD staining of nuclei), and osteogenic on stiff substrate (as indicated by the punctuate CBFα1 staining of nuclei). E, F and G were reproduced from Ref. 82 (© 2004 Rockefeller University Press. Originally published in J. Cell Biol. 166:877–887).

The effects of mechanical forces induced by the surrounding environment (both soluble and insoluble factors) on cell behaviours are becoming an important topic in biological research. Cell adhesion to the ECM regulates cell proliferation and differentiation, and it is believed that tension has been shown to be one of the major mediators of both stimuli. However, most of our understanding of cell functions is based on the studies of cells cultured on stiff surfaces, such as glass coverslips and tissue culture dishes, which are often coated with a very thin layer of ECM. Such a thin ECM coating might not be relevant to the mechanical properties of the microenvironment for most *in vivo* tissues. A previous study has revealed that tissue stiffness runs from very stiff, such as Achilles' tendon (ca. 310 MPa), to very soft, such as mammary glands (ca. 160 Pa) 68. These tissue architectures serve as structural-based scaffolding and a source of inherent forces of mechanical stimulation for single cells. Cellular behaviours such as cell proliferation, differentiation and even apoptosis under stimulation by substrate stiffness are highly tuned 69. Aberrant regulation of *in vivo* tissue stiffness may result in severe and chronic pathological events, such as fibrosis and cancer 70–73. Therefore, understanding cellular responses upon stimulation by mechanical inputs from the substratum or surrounding microenvironment may provide useful information for manipulating cellular behaviours. Several systems have been used to study the influence of substratum stiffness on cellular behaviours. A simple method that is typically used to change the stiffness of a substratum is protein-based ECM gel, such as collagen, fibrin and collagen mixed with fibrin, laminin and other ECM proteins 74–77. Other materials such as polysaccharide-based alginate gel can also be manipulated to exhibit distinct compliance 78, 79. By increasing the protein concentration, the stiffness of the gel can be increased. However, the major disadvantage of using natural gels is that changing the concentration of these natural polymers affects the mechanical stiffness and the ligand density, which may result in uncertain cellular responses upon cell plating on substrates of different stiffness levels. In addition to natural polymers, several groups have also developed synthetic polymers, such as PA and poly(ethylene glycol) (PEG) gels. These gels are chemically inert to cell adhesion unless the surface of the gel is pre-coated with ECM proteins, such as fibronectin or collagen. Thus, the stiffness of the gel can be manipulated by changing the cross-linking of the polymer without changing the material chemistry 43, 44. Several studies have shown that matrix compliance does affect cellular functions. Fusion of myoblasts leads to the formation of polynuclear striated myotubes on collagen strips attached to glass or PA gels with various elasticities. Myotubes exhibit striations only on substrates of intermediate stiffness (ca. 8–10 kPa), but not on substrates of high (17 kPa) or low (ca. 1 kPa) stiffness (Fig. 2C and D) 80. Hepatocytes, as in the case of myotube formation, prefer slightly cross-linked Matrigel that is stiffer than basal Matrigel and can form aggregations and differentiation 81. Using such a tunable substrate system, it is demonstrated that elasticity of the matrix microenvironment can modulate MSC lineage commitment as well. hMSCs differentiate into neuronal-like cells on soft substrate that mimics the stiffness of brain tissues. On the substrate with intermediate stiffness similar to muscles, these cells differentiate into a myoblast lineage, while these cells plated on stiff substrate with a stiffness similar to bone differentiate into osteoblasts 82. In addition, matrix stiffness can modulate soluble factor-induced MSC differentiation. Park *et al*. have found that MSCs on a stiff substrate express smooth muscle cell (SMC) markers, such as α-actin and calponin, whereas MSCs express chondrogenic marker type II collagen and the adipogenic marker, lipoprotein lipase (LPL) on soft substrate. Treatment with transforming growth factor (TGF)-β increases SMC marker expression on stiff substrates, while TGF-β increases chondrogenic marker expression, but suppresses adipogenic marker expression on soft substrates 83. However, the major disadvantage of using synthetic gels is that changing the stiffness of the gel by modulating the cross-linker not only alters the mechanics of these gels but also the material properties, such as the surface porosity, geometry and ligand-binding properties. Therefore, microfabricated, micromolded elastomeric micropost arrays, which decouple the substrate stiffness from adhesive and surface properties to provide a wide range of substrate stiffness values, were reported by Fu *et al*. 84. These devices include the same micropost surface geometry, but differ in post-heights, which can generate substrate stiffness levels across a 1000-fold range. Such devices provide an ECM analogue with different stiffness levels to regulate stem cell commitment and also serve as a force detector to measure contractile forces preceding MSC differentiation at the single cell level 84. Together, these studies provide insights as to how substrate stiffness differentially regulates MSC lineage commitment and how mechanical stimulation cooperates with soluble factors to modulate MSC differentiation.

## Molecular mechanisms relaying substratum stiffness-regulated cellular responses

Although mechanical properties of the matrix affect cell growth and differentiation, how cells sense changes in substrate stiffness and how mechanical signals of substrate compliance are transmitted into cells to regulate cellular behaviours remain to be elucidated. One of the known pathways that mediate stiffness-regulated cell behaviours is through integrin-focal adhesion signalling. A previous study by Shih *et al*. has indicated that stiffer matrix-regulated osteogenesis is mediated by α2-integrin-mediated activation of FAK, Rho-dependent kinase (ROCK) and extracellular signal-regulated kinase (ERK)1/2, given that knockdown of α2-integrin alleviates matrix rigidity–regulated osteogenic outcome and α2-integrin downstream signalling in hMSCs 85. In addition, using tunable PDMS substrates, Wang *et al*. have demonstrated that stiffer substrates promote epidermal cell proliferation, migration and re-epithelialization, whereas softer substrates promote differentiation. The pathway mediating stiffer substrate–induced cell proliferation and migration is through integrin-mediated focal adhesion signalling 86. Other than the current existing signalling pathways, several transcription factors have been implicated as being involved in mechanical force–regulated cell behaviour. Connelly *et al*. have shown that the cell geometry regulates skin stem cell differentiation, where cells plated on smaller islands are more highly differentiated than cells that are allowed to fully spread out 47. This geometry-driven skin stem cell differentiation requires lower expression of stress fibres and a high amount of G-actin, suggesting the involvement of megakaryoblastic leukaemia 1 (MAL) 87 and its binding partner, serum response factor (SRF) 88. The downstream genes JunB (MAL-activated) and FOS are differentially regulated, suggesting that microenvironmental cues, such as chemical and mechanical signals, may work synergistically to regulate cell behaviour through regulating different transcription factors. On the other hand, Dupont *et al*. have demonstrated that the transcription factors, Yes-associated protein (YAP) and transcriptional coactivator with a PDZ-binding motif (TAZ), serve as nuclear relays to mediate mechanical stresses exerted by ECM stiffness and cell shape [89]. The results indicated that RhoA activity and myosin-driven cytoskeletal contractility are required for regulation. YAP and TAZ are also found to be crucial for ECM stiffness– and cell geometry–regulated MSC differentiation, suggesting that YAP and TAZ serve as force sensors to relay mechanical cues raised by the microenvironment.

## Mechanical forces control MSC differentiation

Under physiological conditions, all cells contact the ECM and receive various forces from the surrounding environment; for example, endothelial cells lining blood vessels responding to changes in fluid shear stresses, compression of chondrocytes in knee cartilage and stretching of muscle during muscle contractions. These native mechanical environments of tissues may facilitate the proliferation and differentiation of cells into specific lineages. To address how mechanical forces affect cellular differentiation, several different approaches have been shown to provide robust stimuli for stem cell differentiation. Uda *et al*. have demonstrated that using ECM-coated magnetic beads and applying a magnetic field to generate a twisting force on mouse ESCs resulted in the downregulation of Oct3/4 expression and a decrease in cell proliferation *via* integrin 90. However, the force exerted on the cell–cell adhesion molecule, E-cadherin, has no effect on cell spreading, Oct3/4 expression or self-renewal of mouse ESCs, but significantly increases the cell stiffness. This study suggests that forces exerted on integrin or E-cadherin may act through different force transduction pathways to regulate early embryogenesis. The chondrogenic differentiation of MSCs requires mechanical stimuli, where mechanical loading helped to heal knee articulate cartilage 91 and transplantation of MSCs into the knee joint accompanied by different local mechanical stimuli resulted in different responses in reparative areas 92. To mimic *in vivo* mechanical loading on articulating cartilage, chondrogenesis of MSCs cultured in agarose or collagen with the application of compressive stress has been studied intensively. Compressive forces have been shown to induce expression of type II collagen, aggrecan and chondrogenic-specific transcription factors, such as sox-9 93. Induction of gene expression of TGF-β1 by compressive loading/TGF-β1 treatment suggests that compressive stresses induced chondrogenesis of MSCs by inducing the biosynthesis of TGF-β1 94. In addition to mechanical loading, cyclic stretching has been applied to stem cells to elucidate the role of mechanical stresses on development and differentiation. Cyclic mechanical loading is able to enhance the differentiation of human umbilical cord–derived MSCs into osteoblast-like cells as determined by the expression of osteogenic markers 95, 96. This strain-induced osteogenic differentiation is mediated by the activation of ERK1/2 and a stretch-activated cation channel 96. Furthermore, in a combination 3D culture system, application of mechanical strain to human osteoblastic precursor cells cultured in 3D collagen matrices resulted in increased expressions of osteogenic markers such as cbfa-1, osteopontin, osteocalcin and collagen type I 97. This result suggests that mechanical loading has beneficial effects on tissue generation and may be a good model for tissue engineering of bone and cartilage. Loss of mechanical loading reduces expression of bone-associated markers and osteogenic activity, which leads to bone loss and possibly osteoporosis 98–101.

## Summary

In summary, in addition to soluble factors, mechanical signals also play pivotal roles in regulating stem cell commitment. Mechanical stimuli can either work alone or together with soluble factors to regulate stem cell's fate. These effects are similar to the stem cells in normal *in vivo* conditions where cells receive environmental stimuli to guide differentiation towards specific cells or tissues. Despite the significant progress in the field of mechanical stress on stem cell fate, a number of questions remained unanswered. A recent report proposed that cells may explore environmental changes through stretch-activated ion channels and integrin–cytoskeleton interconnections 37. However, how forces are transduced across cell membranes into cells and connected with intracellular signal pathways to regulate cell behaviours remain to be elucidated.

## Conclusions and future prospects

When in a highly dynamic environment, cells can change their function and reorganize the cytoskeleton in response to both chemical and physical stimuli. Studies of physical stimuli in numerous systems can lead to exploration of mechanical and chemical cues that act on cells and regulate cellular behaviour. It is also true that mechanical signals promote stem cell differentiation into distinct lineages, which mimics the process of embryonic development. However, how MSCs sense and respond to mechanical stimuli remains largely unknown. Several studies have implicated the role of integrins and their downstream signals, and cytoskeletons play important roles in mechanosensing and responding in different types of cells 30, 31, 37, 72, 74. The detailed underlying mechanisms still need to be elucidated. In addition to integrin and its downstream signalling, mechanically sensitive ion channels are also shown to be involved in mechanosensing. These mechanosensitive channels are involved in regulating a variety of cellular functions, including axonal guidance, cell migration, perception of pain and vascular responsiveness 102. Recently, several types of these channels have been identified as mechanosensing channels in mammalian cells, including degenerin/epithelial sodium channel, transient receptor potential channels, acid-sensitive ion channels and others 103. Although some of these channels were shown to be important in maintaining stem cells, the detailed mechanisms of how these channels are involved in mechanosensing and their regulation of stem cell proliferation and differentiation remain to be investigated.

Studies mentioned above indicate that MSCs respond to many mechanical stimuli, such as cyclic stretching, matrix stiffness, compressive stresses and shear stresses, which regulate MSC commitment to specific lineages. Despite a lack of knowledge of how to manipulate MSC fates, these cells are already known to have superior regeneration potential in cell-based therapies when applied to specific environments. However, application of MSCs to cell therapy or tissue engineering requires fully understanding of how to maintain and differentiate MSCs both *in vitro* and *in vivo*. Given that most tissues are highly hierarchically organized, the release of soluble factors and cytokines is controlled in such an organized scaffold with suitable mechanical inputs to control cellular behaviours. In this context, tissue-engineered scaffolds should allow for the establishment of microenvironments that are suitable for specific tissues, such that stem cells/progenitors can proliferate and differentiate into correct cell types for functional tissue regeneration. Thus, by incorporating the idea of mechanical cues together with optimal scaffolds, unlimited progenitor/stem cell sources from autologous sources and novel fabrication methods will enable us to design ideal biomaterials that integrate both biochemical and biophysical cues that mimic the *in vivo* environment for tissue engineering and regenerative medicine. Such cross-disciplinary innovations in tissue engineering and regenerative medicine will allow us to combine stem cell technology to create alternative tissues and injectable/implantable materials to replace and repair damaged parts after ablation of damaged tissues.
